# Standard versus Minimally Invasive Transforaminal Lumbar Interbody Fusion: A Prospective Randomized Study

**DOI:** 10.1155/2017/7236970

**Published:** 2017-06-15

**Authors:** Daniel Serban, Niki Calina, Gabriel Tender

**Affiliations:** ^1^Bagdasar-Arseni Clinical Hospital, Bucharest, Romania; ^2^Louisiana State University, New Orleans, LA, USA

## Abstract

Symptomatic spondylolisthesis patients may benefit from surgical decompression and stabilization. The standard (S) technique is a transforaminal lumbar interbody fusion (TLIF). Newer, minimally invasive (MI) techniques seem to provide similar results with less morbidity. We enrolled patients with at least 6 months of symptoms and image-confirmed low-grade spondylolisthesis, at a single academic institution, between 2011 and 2015. The patients were randomized to either S or MI TLIF. The primary outcome measure was the Oswestry Disability Index (ODI) improvement at 1 year. Secondary outcome measures included length of operation, estimated blood loss, length of hospitalization, and fusion rates at 1 year. Forty patients were enrolled in each group. The differences in mean operative time and estimated blood loss were not statistically significant between the two groups. The patients were discharged after surgery at 4.12 days for the S TLIF group and 1.92 days for the MI TLIF group. The ODI improvement was similar and statistically significant in both groups. The fusion was considered solid in 36 (90%) of patients at 1 year in both groups. In conclusion, the two techniques provided similar clinical and radiological outcomes at 1 year. The patients undergoing MI TLIF had a shorter hospital stay. This trial is registered with NCT03155789.

## 1. Introduction

Surgical management of patients with symptomatic spondylolisthesis has been shown to have superior results compared to nonsurgical treatment [1]. The most common surgical procedure to achieve both decompression of the neural elements and bony stabilization is the standard (S) transforaminal lumbar interbody fusion (TLIF). In recent years, in an attempt to decrease operative morbidity, minimally invasive (MI) techniques have been developed, targeting the same results, but through smaller, paramedian skin incisions. However, the benefits of MI TLIF over the standard technique are yet to be established. In this prospective randomized study, we compared the perioperative and 1-year outcomes in S versus MI TLIF patient groups.

## 2. Methods

### 2.1. Study Design

This prospective randomized study was conducted at “Bagdasar-Arseni” Hospital, the main academic neurosurgical hospital in Bucharest, Romania, between 2011 and 2015. The standardized protocol was approved by the “Bagdasar-Arseni” Hospital Ethics Committee, since the hospital does not have an Institutional Review Board.

### 2.2. Patient Population

All patients were symptomatic with low back pain plus radicular pain and/or neurogenic claudication. Most patients also had sensory changes, and some patients had motor deficits in the affected spinal nerve distribution. All patients had symptoms for at least 6 months and have had attempted conservative treatment, including physical therapy and nonsteroidal anti-inflammatory medication. Magnetic resonance imaging showed low-grade spondylolisthesis (grade 1 or grade 2) at a single lumbar or lumbosacral level. Dynamic X-rays were frequently obtained to evaluate the degree of instability.

The risks and benefits of the two procedures were explained to surgical candidates and they were offered voluntary enrollment in the study. Once the patient was properly worked up and a decision was made that he/she would benefit from a fusion operation, at the clinic visit, the two surgical options were explained to the patient and he/she was asked whether he/she would like to be enrolled in this study. If the answer was “yes,” a call was made to the research coordinator, who blindly extracted the type of surgery to be performed.

At the beginning of the study, 80 identical blocks were marked with either S (standard, *n* = 40) or MIS (minimally invasive, *n* = 40) and mixed in a closed box. When allocation was required, the research coordinator blindly extracted one of these blocks, dictating the type of surgery to be performed.

At the time of enrollment, the fact that, while the costly instrumentation was free of charge to the patient, the study mandated several subsequent clinic visits, with the most important one being at 1 year (at which time imaging would also be performed) was explained to the patients. All patients understood and acknowledged this information at the time of enrollment.

Written informed consent was obtained. All operations were performed or supervised by the senior author (DS).

After the operation, the patients were encouraged to ambulate as soon as pain permitted. The patients were discharged when they met the following criteria: ambulating independently, tolerating oral feedings, voiding normally, and controlling pain with oral medication alone. Postoperative follow-up visits at 3 and 6 months were encouraged. The 1-year visit was mandatory and included obtaining a lumbar computed tomography (CT) without contrast.

### 2.3. Study Interventions

Both the standard and minimally invasive TLIF techniques have been previously described. The following surgical technique variations were used in this study.

#### 2.3.1. Standard TLIF

A midline skin incision was centered on the level of interest. Once the lumbar fascia was encountered, this anatomic plane was followed caudally and slightly lateral to the posterior iliac crest, onto which the fascia was inserted. A small opening in the fascia was made just lateral to the posterior superior iliac spine. A small trephine (1 cm inner diameter) was used to extract a cylinder of cancellous bone, about 3 cm in length, with care not to violate the inner or outer cortex. Occasionally, in patients with relatively preserved disc height, in which a larger amount of graft was anticipated to be needed, a second cylinder was extracted through the same fascial opening. Hemostasis was achieved with bone wax and the fascia was closed with a “figure of 8” 0-Vicryl suture.

Attention was then turned to the midline exposure. The paraspinous muscles were detached subperiosteally and retracted laterally to expose the spinous process and lamina of interest. The dissection was then carried out further laterally to expose the junction between the lateral facet and transverse process (or ala, for the sacrum). The operative microscope was brought into the field and a hemilaminectomy and facetectomy were performed on the side of the worst symptoms, up to the level of the yellow ligament's cranial insertion. The tip of the lateral facet was then removed with the high-speed drill, down to the cranial aspect of the caudal pedicle. At this time, the yellow ligament was removed with Kerrison Rongeurs. If central or severe contralateral stenosis was noted on preoperative films, the remainder of the ipsilateral lamina was drilled off to the bottom of the spinous process and the yellow ligament removal was extended under the spinous process to the contralateral side. Once the canal and foraminal decompression was complete, attention was turned back to the ipsilateral epidural veins, which were coagulated and sharply transected. The annulus of the disc of interest was exposed. Occasionally, the exiting nerve root was identified in the foramen, particularly in patients with grade 2 spondylolisthesis. A standard discectomy and endplate preparation was then performed, with minimal medial retraction of the dural sac, if necessary. The ideal height for the cage was evaluated by inserting increasing size trials under fluoroscopic guidance. Morselized iliac crest graft material was then inserted in the interspace and impacted. The adequate size polyether ether ketone (PEEK) cage, also filled with autograft material, was then inserted in the interspace as far anteriorly as possible, attempting to cross the midline. The pedicle screws were then inserted bilaterally by starting at the junction of the transverse process, lateral facet, and pars interarticularis (or ala and facet, for the sacrum) and then advancing in a lateral to medial and cranial to caudal fashion. The construct was then locked in place with the adequate length rod and set screws. While no dedicated efforts were made to reduce the spondylolisthesis, most cases resulted in at least a one grade reduction, just by opening up the disc space and placing the screw-rod construct. The wound was closed in anatomical layers over a subfascial drain.

#### 2.3.2. Minimally Invasive TLIF 

Two paramedian skin incisions were made, about 3 cm in length and about 5 cm off midline, centered on the level of interest, as determined by fluoroscopy. For the L4-L5 or L5-S1 levels, the incisions were in identical position and located just cranial to the iliac crest. Once the lumbar fascia was encountered, its insertion onto the iliac crest was just caudal to the skin incision. The posterior superior iliac spine was palpated and a small fascial opening was made just lateral to it. The same trephine was used to extract one or two cylinders of cancellous bone, followed by hemostasis and fascial closure with a “figure of 8” 0-Vicryl suture. See Operative Video in Supplementary Material available online at https://doi.org/10.1155/2017/7236970.

Attention was then turned to the TLIF exposure. The lumbar fascia was opened medial to the skin incision, and the paraspinous muscles were bluntly spread with dilating tubes of increasing diameter. A final tubular retractor was docked on the pars interarticularis of interest, with the medial border of the retractor resting against the base of the spinous process and the caudal border just below the caudal edge of the lamina of interest. The tubular retractor was positioned in a lateral to medial direction, usually at 30 to 45 degrees. The operative microscope was brought into the field at this time. Using the high-speed drill, a laminectomy, medial facetectomy, and partial lateral facetectomy (removal of the rostral aspect of the lateral facet, down to the cranial edge of the pedicle) were then performed, similar to the open technique. The yellow ligament removal was performed ipsilateral or bilateral, under the spinous process, depending on the degree of central and contralateral foraminal stenosis. After coagulation of the ipsilateral epidural veins, a standard discectomy and endplate preparation was performed. The angle of the retractor facilitated a better contralateral discectomy (under the spinal canal and posterior longitudinal ligament) and less need for medial retraction of the dural sac. Autograft material was then impacted in the interspace, followed by insertion of a PEEK interbody cage under fluoroscopic guidance.

After this, the retractor's angle was changed slightly towards the lateral aspect of the exposure, in order to see the junction between the lateral facet and transverse process (or facet and ala, for the sacrum). The entry point for the screws was started with the high-speed drill and then the path for the screws was made with pedicle finders, through the retractor tube, and under fluoroscopic guidance. K-wires were placed in the created paths, and then the retractor tube was removed and the pedicle screws were inserted over the K-wires in percutaneous-like fashion. The opposite side screws were inserted percutaneously and then the rods and set screws were used to complete the construct. The wounds were closed in anatomical layers.

### 2.4. Study Measures

The primary outcome measure was the ODI improvement at 1 year from surgery. The secondary outcomes included perioperative parameters (operative time, estimated blood loss, and postsurgical days in the hospital) and the fusion rates at 1 year. The fusion was assessed by CT at 1 year, by an independent radiologist, according to a 4-grade CT-based classification [2] (Grade I: complete fusion, Grade II: partial fusion, Grade III: unipolar pseudarthrosis, and Grade IV: bipolar pseudarthrosis).

### 2.5. Statistical Analysis

Data are presented as mean ± SD. The paired *t*-test was used to analyze the ODI scores before and at 1 year after surgery, and the independent-samples *t*-test was used to compare the indicators between the two groups. The chi-square test was used to analyze nonparametric factors, such as fusion rates. A *p* value < 0.05 was considered statistically significant.

## 3. Results

A total of 80 patients were enrolled, with 40 patients in each group.

Table 1 shows the patient demographics in the two groups. The groups were similar in age, gender, and clinical characteristics. The age was 50.12 ± 11.09 years in the S TLIF group and 51.3 ± 9.36 years in the MI TLIF group. There were 23 and 24 females in the S and MI TLIF group, respectively. Work status was similar in both groups. The body mass index was 29.92 ± 5.7 in the standard and 28.97 ± 5.18 in the MI TLIF group. The average duration of symptoms was 2.46 ± 1.34 and 2.32 ± 1.52 years in the S and MI TLIF groups, respectively. In the S TLIF group, there were 2 L3-4, 14 L4-5, and 24 L5-S1 levels treated, with 20 grade 1 and 20 grade 2 spondylolistheses. In the MI TLIF group, there were 2 L3-4, 15 L4-5, and 23 L5-S1 levels treated, with 19 grade 1 and 21 grade 2 spondylolistheses. There were 12 and 14 isthmic spondylolistheses in the S and MI TLIF groups, respectively, with the remainder being degenerative (Table 2).

The mean operative time was 296 ± 101 minutes in the S TLIF group and 321 ± 85 minutes in the MI TLIF group (*p* = 0.21). The mean estimated blood loss was 417 ± 211 ml in the S TLIF group and 351 ± 198 ml in the MI TLIF group (*p* = 0.15). There were 4 transfusions in the S TLIF and 3 transfusions in the MI TLIF group. The mean number of days from surgery to discharge was 4.12 ± 0.88 in the standard TLIF group and 1.92 ± 0.52 in the MI TLIF group (*p* < 0.0001). The ODI improved from 38 ± 7 to 11 ± 6 in the S TLIF group (ODI difference: 26 ± 7, *p* < 0.0001) and from 37 ± 6 to 11 ± 6 in the MI TLIF group (ODI difference: 26 ± 8, *p* < 0.0001) (*p* = 0.96). In both groups, the fusion was considered solid (Grade I) in 36 (90%) and partial (Grade II) in 4 (10%) patients at 1 year. There were no reoperations for pseudarthrosis or any other postoperative complication.

There were 2 superficial wound infections in the standard TLIF group, which resolved with oral antibiotic treatment alone.

## 4. Discussion

Romania (population: approx. 20 million) has a “centralized medicine” system, in which patients from the entire country are referred to Bucharest (the capital, population: approx. 2 million) and 3 other smaller centers/cities for specialty surgical care. The Romanian population is homogenous in multiple ways: 97% white, over 80% Easter Orthodox, and a predominant middle class society. There is minimal welfare/disability support (i.e., no work = no pay) and the usage of oral narcotic pain medication is virtually inexistent (patients are encouraged to take NSAIDs for pain). Overall, people are typically compliant with “doctor's recommendations.”

The “Bagdasar-Arseni” hospital is the main neurosurgical hospital in Bucharest and has the most advanced spinal surgery department. About 7000 patients with lumbar pathology were evaluated at the “Bagdasar-Arseni” hospital Spine Department between 2011 and 2015. The exact information and charts of these patients, as well as those not meeting the inclusion criteria and the ones who declined to participate, were not saved nor kept separate to be analyzed in the study, due to logistical difficulties (large number of patients versus limited research personnel). Therefore, an accurate CONSORT flow diagram could not be reliably created.

The minimally invasive TLIF technique has been described over 10 years ago [3]. However, prospective randomized studies comparing the outcomes of standard versus minimally invasive TLIF are scarce [4]. Moreover, meta-analyses of the available literature have yielded conflicting conclusions [5, 6].

Most comparative studies currently available are retrospective [7–10]. Wong et al. [10] compared 144 MI TLIF with 54 standard TLIF patients and found statistically significant benefits for MI TLIF in operating room time, blood loss, primary infection rates, secondary medical complications, LOS, transfusion need, hospital costs, short-term and long-term pain outcomes, and short-term and long-term ODI scores. Terman et al. [8] retrospectively reviewed his results at a single institution in obese patients undergoing MI TLIF (*n* = 53) and S TLIF (*n* = 21) and concluded that both procedures had good results, similar to those in nonobese patients. Other studies with fewer patients enrolled had results generally favoring the MI TLIF technique.

Several prospective nonrandomized studies addressed the same topic. Parker et al. [4] evaluated 100 patients (50 in each group) for both clinical outcomes and cost-savings. The MI TLIF patients did significantly better in terms of operative blood loss, hospital stay, 2-year cost, and return to work. The two groups were similar in terms of surgical morbidity and short- and long-term clinical effectiveness.

The only prospective randomized study we could identify was published by Wang et al. [11]. The authors evaluated 41 patients in the MI TLIF and 38 patients in the S TLIF group. They concluded that MI TLIF patients have less sacrospinalis muscle injury, resulting in early functional recovery and superior short-term treatment effects. However, they found similar long-term efficacy for the two groups.

Our study prospectively evaluated 80 patients with symptomatic lumbar spondylolisthesis who were randomized to either standard or minimally invasive TLIF. The two groups were similar in demographic and clinical characteristics and represent a fairly typical Romanian population. While the patients understood the risks and benefits of each technique, there was commonly no patient preference of one technique versus the other. Moreover, likely due to the centralized medicine system, there was very little noncompliance in terms of questionnaire completion and follow-up visits. No patients were lost to follow-up, likely due to the centralized medicine system and the patient signed agreement, at the enrollment in the study, that stipulated free-of-charge instrumentation and mandatory follow-up visits and imaging.

The results of our study are not surprising, although not entirely aligned with most other studies in the literature (Table 3). The main finding is that the primary outcome, the ODI improvement at 1-year postoperatively, was clinically and statistically significant, but similar between the two groups. The operative time was longer in the MI TLIF group, likely due to the learning curve and novelty of the technique. The estimated blood loss was slightly less in the MI TLIF group; however, it was higher than most results reported in the literature. The time to discharge was significantly shorter for patients in the MI TLIF group (mean: 1.92 days) compared to the S TLIF group (mean: 4.12 days). This is consistent with other studies and may imply reduced costs, although cost was not analyzed in our study. The fusion rate was high and similar in both groups, consistent with other reports.

Limitations of this study include the relatively low number of patients enrolled and the fact that the study was performed at a single institution.

## 5. Conclusion

In this prospective randomized study comparing S and MI TLIF in patients with symptomatic spondylolisthesis, the MI TLIF group patients had significantly shorter hospitalization than the S TLIF group patients. However, the 1-year clinical and radiological outcomes were similar in both groups.

## Supplementary Material

The operative video illustrates the surgical steps to perform a minimally invasive transforaminal lumbar interbody fusion.

## Figures and Tables

**Table 1:** Patient demographics [S TLIF = standard transforaminal lumbar interbody fusion; MI TLIF = minimally invasive transforaminal lumbar interbody fusion; SD = standard deviation; BMI = body mass index; F = female; M = male].

	S TLIF (SD; range)	MI TLIF (SD; range)
Age	50.12 (11.09; 17–67)	51.3 (9.36; 34–69)
Sex	23 F/17 M	24 F/16 M
BMI	29.92 (5.7; 19–44)	28.97 (5.18; 21–40)

**Table 2:** Spondylolisthesis information [S TLIF = standard transforaminal lumbar interbody fusion; MI TLIF = minimally invasive transforaminal lumbar interbody fusion; Degen = degenerative].

	S TLIF	MI TLIF
Level		
L3-4	2 (5%)	2 (5%)
L4-5	14 (35%)	15 (37.5%)
L5-S1	24 (60%)	23 (57.5%)
Type		
Degen	28 (70%)	26 (65%)
Isthmic	12 (30%)	14 (35%)
Grade		
1	20 (50%)	19 (47.5%)
2	20 (50%)	21 (52.5%)

**Table 3:** Comparison of clinical and radiological outcomes [S TLIF = standard transforaminal lumbar interbody fusion; MI TLIF = minimally invasive transforaminal lumbar interbody fusion; EBL = estimated blood loss; POD discharge = postoperative day of discharge].

	S TLIF (SD; range)	MI TLIF (SD; range)	*p* value
ODI preop	38.35 (7.58; 26–57)	37.75 (6.59; 29–60)	0.707
ODI postop 1 Y	11.67 (6.09; 2–24)	11.52 (6.56; 2–23)	0.995
ODI difference	26.62 (7.75; 12–45)	26.47 (8.17; 11–45)	0.962
Operative time	296.22 (101.01; 116–493)	321.92 (85.57; 212–473)	0.219
EBL	417.5 (211.69; 150–1100)	351.25 (198.87; 100–1000)	0.153
POD discharge	4.12 (0.88; 2–6)	1.92 (0.52; 1–3)	<0.0001
Fusion at 1 y	90%	90%	1
